# Prostaglanin-E2 Potentiates the Suppressive Functions of Human Mononuclear Myeloid-Derived Suppressor Cells and Increases Their Capacity to Expand IL-10-Producing Regulatory T Cell Subsets

**DOI:** 10.3389/fimmu.2019.00475

**Published:** 2019-03-18

**Authors:** Sergej Tomić, Bojan Joksimović, Marina Bekić, Miloš Vasiljević, Marijana Milanović, Miodrag Čolić, Dragana Vučević

**Affiliations:** ^1^Department for Immunology and Immunoparasitology, Institute for the Application of Nuclear Energy, University of Belgrade, Belgrade, Serbia; ^2^Medical Faculty of the Military Medical Academy, University of Defense in Belgrade, Belgrade, Serbia; ^3^Medical Faculty Foča, University of East Sarajevo, Lukavica, Bosnia and Herzegovina

**Keywords:** myeloid derived suppressor cells, prostaglandin E2, type 1 regulatory T cells, FoxP3^+^ regulatory T cells, checkpoint blockade

## Abstract

Myeloid-derived suppressor cells (MDSC) emerged as major factors driving the tumor progression due to numerous immunosuppressive mechanisms they possess. Prostaglandin (PG)E2 is shown critical for the induction of MDSC and their suppressive functions *in vivo*, but it is poorly understood how it affects the capacity of MDSC to induce different subsets of regulatory T cells (Treg). By using a novel protocol for the generation of mononuclear (M)-MDSC, we showed that PGE2 potentiates the GM-CSF/IL-6-dependent induction of CD33^+^CD11b^+^HLA-DR^−^CD14^+^ M-MDSC *in vitro*. PGE2 diminished the capacity of GM-CSF/IL-6 M-MDSC to produce proinflammatory cytokines upon activation and augmented their capacity to produce IL-27, IL-33, and TGF-β. These results correlated with an increased potential of GM-CSF/IL-6/PGE2 M-MDSC to suppress T cell proliferation, expand alloreactive Th2 cells, and reduce the development of alloreactive Th17 and cytotoxic T cells. Interestingly, GM-CSF/IL-6/PGE2 M-MDSC displayed a lower capacity to induce TGF-β-producing FoxP3^+^ regulatory Treg compared to GM-CSF/IL-6 M-MDSC, as a consequence of reduced IDO-1 expression. In contrast, GM-CSF/IL-6/PGE2 M-MDSC potentiated IL-10 production by CD8^+^T, Th2, and particularly CD4^+^FoxP3^−^ type 1 Treg, the latter of which depended on ILT3 and ILT4 expression. Cumulatively, PGE2 potentiated the suppressive phenotype and functions of GM-CSF/IL-6-induced M-MDSC and changed the mechanisms involved in Treg induction, which could be important for investigating new therapeutic strategies focused on MDSC-related effects in tumors and autoimmune diseases.

## Introduction

Cancer immunotherapy has been improved significantly by the discovery of checkpoint inhibitors targeting cytotoxic T leukocyte antigen (CTLA)-4 and programmed death (PD)-1 axis. Yet, one-third of cancer patients receiving checkpoint inhibitors relapse, and the mechanisms for resistance acquiring are poorly understood (1). Myeloid-derived suppressor cells (MDSC), which are heterogeneous cell population present in virtually all individuals with a diagnosed tumor (2, 3), have been recognized as major suppressors of the anti-tumor response, and a major limiting factor for the efficacy of checkpoint inhibition therapy (2, 4, 5). MDSC promote neoangiogenesis and tumor metastasis, by producing VEGF and metalloproteases, respectively (6). More importantly, MDSC utilize different suppressive mechanisms to limit the activation of immune cells, particularly of cytotoxic T cells (7), which are the major effector cells in anti-tumor response (8). Two major subtypes of MDSC were described in humans, both lacking lineage markers expression (CD3, CD19, CD20, CD56) and HLA-DR, while expressing myeloid markers CD33 and CD11b. The mononuclear subtype (M-MDSC) express a monocytic CD14 marker, whereas polymorphonuclear subtype (PMN-MDSC) express CD15 (9). Although these subtypes display different suppressive mechanisms, the studies on tumor models in mice suggested that M-MDSC exhibit a stronger immunosuppressive potential compared to PMN-MDSC (10). Additionally, a preferential accumulation of M-MDSC in the tumor of melanoma and prostate cancer patients (3, 11), suggests that M-MDSC are the key factors of immune suppression in some types of cancer. Besides tumor, M-MDSC appear an important factor in other chronic inflammatory processes, such as autoimmunity (12). From the clinical perspective, providing MDSC or their products may improve the efficacy of therapies for several autoimmune diseases (13). Moreover, MDSC are being advocated as promising therapeutic strategy in organ transplantation along with other myeloid suppressor cells, such as regulatory macrophages and tolerogenic dendritic cells (DC) (14). In line with this, it was shown that the induction of tolerance to kidney, skin and cardiac allografts is associated with infiltration of grafts by MDSC (15, 16).

Direct immunosuppressive mechanisms of M-MDSC have been studied extensively (7), and they include induction of M2 macrophages (17), suppression of NK cell-mediated cytotoxicity (18), and suppression of T cell activation by depletion of essential amino acids, such as arginine, tryptophan, and cysteine (19–21). In addition, M-MDSC were shown to recruit regulatory T cells (Treg) in the tumor (22), and promote *de novo* induction of FoxP3^+^ Treg (23), thus spreading the immune suppression further. Different mechanisms were described to contribute their capacity to induce Treg, including the involvement of CD80, TGF-β (24), PD1L (25), IDO-1 (26), ILT-3 (27), and ILT-4 (7, 28). However, it has been shown that these molecules are also involved in the induction of non-conventional Treg subsets, such as suppressor CD8^+^ and type 1 regulatory T (Tr-1) cells (29, 30). Our previous findings confirmed these mechanisms as well (31, 32). Moreover, non-conventional Treg subsets were shown to exhibit even stronger suppressive effects than the conventional FoxP3^+^ Treg (33, 34). However, it remained unclear whether M-MDSC induce Treg other than FoxP3^+^, and which mechanisms are involved in their induction.

Detailed analyses of Treg-inducing mechanisms by human M-MDSC, enabling the development of new immunotherapeutic strategies in cancer and autoimmune diseases, is partially hampered by their relatively short half *in vivo*, and *in vitro* upon isolation (7). Consequently, several *in vitro* protocols have been proposed for the generation of M-MDSC *in vitro* (35–37). It was suggested that M-MDSC could be differentiated from monocytes *in vitro* by using GM-CSF and IL-4 in the presence of PGE2 (36, 38) or IL-10 (37), which shift the differentiation of monocytes away from DC, toward M-MDSC-like cells. However, similar protocols were described for the induction of tolerogenic DC (39, 40). To limit these controversies, Bronte et al. (9) suggested minimal phenotypic and functional criteria for defining M-MDSC. However, the majority of reported data did not show clearly whether the phenotypic and functional properties of obtained M-MDSC comply with these criteria. Lechner et al. (35) suggested that GM-CSF and IL-6 are the most potent cytokines for the induction of M-MDSC within PBMC, but the phenotypic and functional properties of these cells resembled more to PMN-MDSC. So, it remained unclear whether M-MDSC could be differentiated by using GM-CSF and IL-6. GM-CSF was demonstrated as a critical factor to maintain the myeloid cell viability in cancer (41), and IL-6 was shown as the most potent proinflammatory cytokine linked to MDSC accumulation and consequent tumor progression (42, 43). Besides, PGE2, and cyclooxygenase 2 (COX2) overexpression were shown critical for the differentiation of MDSC from mice bone marrow and tumor progression in animal models (44). Additionally, PGE2 was shown to induce M-MDSC (18, 45) and potentiate their suppressive properties in cancer patients (46), but no data reported how it affects the capacity of M-MDSC to induce different Treg subsets. Taking into account *in vivo* data on the importance of these inflammatory mediators, we hypothesized that the combination of GM-CSF and IL-6 enables the differentiation of M-MDSC from human monocytes and that PGE2 significantly potentiates their suppressive phenotype and functions *in vitro*. By doing so, PGE2 alters the capacity of M-MDSC to induce different subtypes of Treg. According the Minimum Information about Tolerogenic Antigen-presenting cells (MITAP) (47), the hypothesis was tested by analyzing viability, phenotype, cytokines production, suppressive capacities, and the mechanisms involved in the induction of Treg.

## Materials and Methods

### Cells

All experiments involving human blood samples were approved by the Ethical Board of the Military Medical Academy, University of Defense (MMA), and carried out in accordance with the MMA Guidelines. PBMC were obtained from buffy coats of healthy volunteers, who signed the Informed Consent in accordance with the Declaration of Helsinki, using density gradient centrifugation on lymphocyte separation medium 1077 (PAA, Linz, Austria). CD14^+^ monocytes and CD3^+^ T cells were isolated from PBMC with magnetic-activated cell sorting (MACS) of untouched cell populations, by using the Monocyte Isolation Kit II and Pan T cell isolation kit (Miltenyi Biotec, Bergisch Gladbach, Germany), respectively. The purity of CD14^+^ monocytes and CD3^+^ T cells was higher than 85 and 95%, respectively, as evaluated by flow cytometry (Cube 6, Sysmex Partec GmbH, Görlitz, Germany; BD LSR II, San Jose, CA, USA).

Monocytes (1 × 10^6^/mL) were cultivated in CellGenix® GMP Dendritic Cell Medium (CellGenix, Freiburg, Germany) supplemented with 100 ng/mL of human recombinant granulocyte macrophages colony stimulating factor (GM-CSF; Novartis, Basel, Switzerland) and 20 ng/mL of human recombinant IL-4 (Roche Diagnostics, Basel, Switzerland) to induce immature DC. In some experiments, PGE2 (1 μg/ml, Sigma Aldrich Co.) was supplemented to the GM-CSF/IL-4 medium from the beginning of cells' cultivation to obtain GM-CSF/IL-4/PGE2 M-MDSC. The monocytes of the same donors were differentiated in the presence of GM-CSF (100 ng/mL) and human recombinant IL-6 (40 ng/ml, R&D Systems, Minneapolis, MN, USA) to obtain GM-CSF/IL-6 M-MDSC, or additionally with PGE2, to obtain GM-CSF/IL-6/PGE2 M-MDSC. After 5 days of cultivation at 37°C, 90% humidity, and 5% CO_2_, the cells were either stimulated for the next 16 h with 50 ng/mL of interferon (IFN)-γ (R&D Systems, Minneapolis, MN, USA) and 200 ng/mL of LPS from Escherichia coli 0.111:B4 (Sigma-Aldrich Co.), or left unstimulated. Subsequently, the cells were analyzed for their phenotype or used in the functional assays with T cells, whereas cell-free supernatants were used for determination of the cytokines' levels.

### Mixed Cell Cultures

Before the co-cultivation with allogeneic PBMC or T cells, M-MDSC were washed twice in RPMI medium to prevent the transfer of cytokines and stimuli. The capacity of M-MDSC and DC to suppress the proliferation of PBMC was tested by co-cultivating M-MDSC and DC (2.5–0.62 × 10^4^/well) with allogeneic PBMC (2 × 10^5^/well) in the presence of phytohemagglutinin (10 μg/ml, Sigma Aldrich) for 5 days. Prior to the test, PBMC were labeled with carboxyfluorescein succinimidyl ester (CFSE, 0.5–2 μM, Thermo Fisher Scientific, Waltham, MA, USA) according to manufacturer's protocol. The suppressive effect of M-MDSC on T cell proliferation was tested by co-cultivating M-MDSC (2.5 × 0.62 × 10^4^/well) and MACS-purified allogeneic CFSE-labeled T cells (1 × 10^5^/well), in the presence of plate-coated anti-CD3 Ab (5 μg/ml, R&D Systems) and soluble anti-CD28 Ab (1 μg/ml, Miltenyi Biotec, Bergisch Gladbach, Germany). The proliferation of alloreactive T cells was tested in the absence of CD3/CD28 stimulation by co-cultivating LPS/IFN-γ-treated M-MDSC or DC with allogeneic CFSE-labeled T cells (1 × 10^5^/well) or T cells alone, for 5 days. To induce Treg, allogeneic T cells (1 × 10^5^) were cultivated alone, or in the presence of LPS/IFN-γ-treated M-MDSC (2 × 10^3^/well) for 3 days, followed by 3-day stimulation with IL-2 (3 ng/ml, R&D Systems), as described previously for tolerogenic DC (31, 32). To assess the mechanisms of Treg induction, some M-MDSC/T cell co-cultures were supplemented with IDO-1 inhibitor 1-methyl-tryptophan (1-MT, 0.3 mM; Sigma-Aldrich Co.), blocking anti-ILT-3 or anti-ILT-4 Ab (both at 2 μg/mL; R&D Systems), or isotype control Ab (anti-rat IgG2b; Thermo Fisher Scientific). For cytokines' analysis in M-MDSC/T cell cocultures, the cultures were treated with PMA (20 ng/mL) and ionomycin (500 ng/mL) (both from Sigma-Aldrich Co.) for the last 4 h before harvesting the cell-free supernatants. For the flow cytometric detection of intracellular cytokines in T cells, the co-cultures were treated with PMA/ionomycin and monensin (3 μM; Sigma-Aldrich Co.) for the last 3 h of incubation.

### Proliferation, Viability, and Cytokine Production

The proliferation of allogeneic CFSE-labeled PBMC and T cells co-cultivated with DC and M-MDSC, was analyzed by flow cytometry within the gated propidium (PI)^−^ population, by measuring CFSE dilution during the cells' divisions, as described (32). The percentage of proliferation was calculated using the proliferation fit statistics in FCS Express 4 software (*De Novo* Software, Glendale, CA, USA). The relative proliferation in suppression assays was calculated as the percentage of proliferation relative to control (i.e., without the presence of DC or M-MDSC, 100%). The apoptosis of M-MDSC and viability/cell count of T cells after the co-cultures with allogeneic M-MDSC was determined by staining the cells with Muse® Annexin V and Dead Cell Assay Kit and Muse® Count &Viability Assay Kit, respectively, followed by the analysis on Muse Cell Analyzer (Merk Millipore, Wien, Austria). The cytokine concentrations in cell culture supernatants were determined by appropriate ELISA kits (R&D Systems) spectrophotometrically, and bead-based immunoassays (Biolegend, San Diego, CA, USA) by flow cytometry.

### Flow Cytometry

The phenotype analysis of M-MDSC, DC, and T cells was carried out by flow cytometry after staining the cells with the fluorescently labeled Abs (Clone) and reagents: IgG1 negative control-PE (MCA928PE), IgG1 negative control-FITC (MCA928F) (Bio-Rad); anti-CD1a-PerCP/Cy5.5 (HI149), anti-HLA-DR-APC/Cy7 (L234), anti-CD80-APC (2D10), anti-IL-4-PerCP/Cy5.5 (MP4-25D2), anti-IL-4-PE (42D1), anti-ILT-4-APC, anti-CD56-PerCP/Cy5.5 (MEM-188), anti-CD19-PerCP/Cy5.5 (HIB19), anti-CD25-PE (BC96), anti-CD25-PerCP/Cy5.5 (M-A251), anti-CD127-PE (A019D5), anti-CD11b-PE, anti-CD11b-Pe/Cy7 (ICRF44), anti-IL-10-APC, anti-IL-10-PE (JES5-16E3), anti-TGF-β-APC (TW4-6H10), anti-IL17A-Alexa Fluor 488 (BL168), anti-IFN-γ-APC, anti-IFN-γ-FITC (4S.B3), IgG1 negative control-PerCP/Cy5.5 (HTK888), anti-CD73-PerCP-Cy5.5 (AD2) (all from Biolegend); anti-HLA-DR PerCP (L243), anti-IDO-1-APC (700838), anti-CD33-APC (6C5/2), anti-CD4-FITC, anti-CD4-APC (11830), anti-TGF-β-PE (9016) (all from R&D Systems), anti-CD14-FITC (TUK4) (Miltenyi Biotec), anti-CD86-PE (IT2.2), streptavidin-PerCP, streptavidin APC, anti-ILT3-PE (ZM4.1), anti-CD209-FITC (eB-h209), anti-CD206-APC (19.2), anti-CCR7-FITC (3D12), IgG1 negative control APC (MA5-18093), anti-CD39-PE (eBioA1), anti-IL-17A-APC (eBio17B7) (all from Thermo Fisher); anti CD40- APC (5C3), anti-IL-12 (p40/p70)-PE (C11.5), anti-CD3-PE (SK7), anti-FoxP3-PerCP/Cy5.5, anti-FoxP3-Alexa Fluor 488 (236A/E7) (all from BD Pharmingen, San Diego, CA, USA), anti-CD8-PerCP/Cy5.5 (HIT8a) (Elabscience), and anti-CD4-PE (MEM-241) (Partec Sysmex). Surface staining with primary Abs was conducted in PBS/0.1% NaN3/0.5% FBS prior to intracellular staining that was carried out using the BD fixation/permeabilization kit (Becton Dickinson). The gates for cultivated M-MDSC and T cells were set according to their specific forward scatter (FS) and side scatter (SS) properties, thereby avoiding dead cells with low FS/SS signal. The gates, containing more than 97% of live cells, were confirmed by independent PI staining of non-permeabilized cells. The signal overlap between the fluorescent channels was compensated before each experiment using the single-labeled samples. The non-specific fluorescence was determined by using the appropriate isotype control Abs and fluorescence minus one/two controls (FMO). The number of cytokine-producing cells was calculated according to the number of viable cells and the percentages of cytokine-producing cells detected after the co-cultures by flow cytometry.

### Statistical Analysis

The results are presented as representative data or as mean ± SD values of at least three independent experiments carried out with cells of different healthy donors. The differences between the treatments were analyzed by repeated measures (RM) ANOVA with Tukey's multiple comparison test or paired *T*-test, using the GraphPad Prism software (GraphPad, La Jolla, CA, USA). All tests were two-sided with the significance level of *p* = 0.05.

## Results

Considering the important role of GM-CSF, IL-6 and PGE2 in M-MDSC induction and functions *in vivo* (41–46), we first sought to establish the model for the generation of M-MDSC from monocytes *in vitro* based on these factors. The phenotypic and functional properties of M-MDSC were assessed according to the criteria proposed by Bronte et al. (9). As a control, we also used the protocol for M-MDSC differentiation based on GM-CSF/IL-4/PGE2 (48), and as a negative control, we used GM-CSF and IL-4, which induce immature DC.

### PGE2 Potentiates GM-CSF/IL-6-Mediated Induction of M-MDSC Phenotype

**Figure 1:**
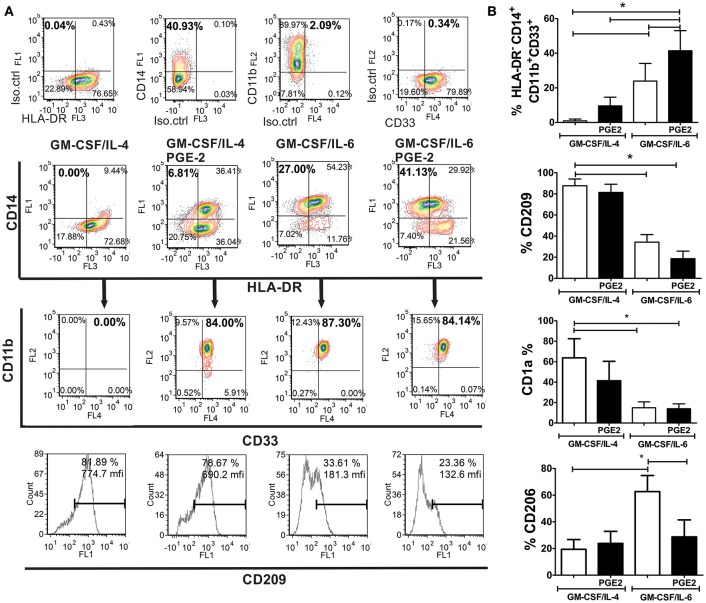
Phenotypic analysis of M-MDSC and DC generated from human monocytes *in vitro*. **(A,B)** The monocytes were cultivated in the presence of GM-CSF/IL-4, GM-CSF/IL-4/PGE2, GM-CSF/IL-6, or GM-CSF/IL-6/PGE2 for 5 days, followed by their phenotypic analysis. **(A)** A representative analysis of the co-expression of HLA-DR, CD14, CD11b, and CD33 is shown. The doublets and the dead (FSC^low^) cells were gated-out (not shown), and the quadrants were set according to the single-labeled samples (first row). CD11b/CD33 plots were gated from the HLA-DR^−^CD14^+^ region, and the percentage of HLA-DR^−^CD14^+^CD33^+^CD11b^+^ was calculated based on these plots. The expression of CD209 was analyzed within the total gated cell population. **(B)** The summarized results on % of HLA-DR^−^CD14^+^CD33^+^CD11b^+^ cells, CD209^+^, CD1a^+^, and CD206^+^ are shown as mean ± SD from 4 independent experiments carried out with different donors. ^*^p < 0.05 between the indicated samples (RM ANOVA, Tukey post-test).

MACS-sorted CD14^+^ monocytes from healthy donors that were used for differentiation, contained <2 % of HLA-DR^−^ CD14^+^ CD33^+^ CD11b^+^ SSC^low^ (M-MDSC) and HLA-DR^−^CD15^+^ CD33^+^ CD11b^+^ SSC^low^ (PMN-MDSC) cells, as expected for healthy donors (9). After their differentiation with GM-CSF and IL-6, up to 34% (23.9 ± 10.2%) of cells showed HLA-DR^−^CD14^+^CD33^+^CD11b^+^ M-MDSC phenotype (Figure 1A), and no significant percentage of CD15^+^ cells was present in the population (data not shown). The addition of PGE2 to the GM-CSF/IL-6 cocktail induced a significantly higher percentage of HLA-DR^−^CD14^+^CD33^+^CD11b^+^ M-MDSC cells (41.23 ± 11.6%) compared to GM-CSF/IL-6 (Figures 1A,B). GM-CSF/IL-4 induced almost complete down-regulation of CD14 on immature DC and no significant percentage of HLA-DR^−^CD14^+^CD11b^+^CD33^+^ was detected. The addition of PGE2 to the GM-CSF/IL-4 cocktail induced a significantly higher percentage of CD14^+^ cells and majority of the cells expressed HLA-DR as well. The percentage of HLA-DR^−^CD14^+^CD33^+^CD11b^+^ M-MDSC was not higher than 15% (9.5 ± 5.0%) (Figure 1A). Besides, GM-CSF/IL-4-based protocols induced differentiation of CD209^+^ cells predominantly, unlike GM-CSF/IL-6-based protocols. The expression of CD1a was most prominent on immature DC, whereas other cell types displayed lower expression of this molecule, and the statistically significant reduction of CD1a was detected only in GM-CSF/IL-6-based protocols. Interestingly, the M-MDSC differentiated with GM-CSF/IL-6 alone, displayed significantly higher expression of CD206 compared to other tested cells. These results suggested that, according to the phenotypic criteria for M-MDSC (9), GM-CSF/IL-6-based protocols were more potent at inducing M-MDSC, and that PGE2 significantly potentiated this effect.

### PGE2 Potentiates Suppressive Functions of M-MDSC

**Figure 2:**
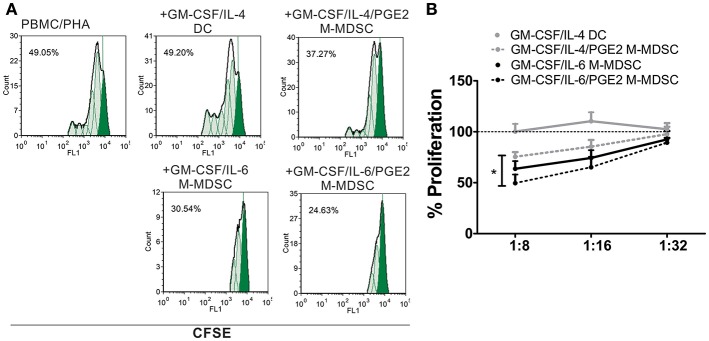
Suppressive capacity of M-MDSC and DC. **(A,B)** The PHA-stimulated CFSE-labeled allogeneic PBMC (2 × 10^5^/well) were co-cultivated with M-MDSC or DC at different cell ratios (1:8–1:32, M-MDSC:T cells) for 5 days, followed by flow cytometry analysis. **(A)** A representative analysis of PBMC proliferation is shown with the G0 generation marked in full green. The % of proliferation was calculated by using the proliferation fit statistics. **(B)** The summarized results are shown as the mean relative proliferation % ± SD (*n* = 3), i.e., % proliferation of control PBMC/PHA cultures in each experiment (100%). All M-MDSC suppressed the proliferation of PBMC at 1:8 cell ratio (not labeled). ^*^p < 0.05 GM-CSF/IL-4/PGE2 M-MDSC vs. GM-CSF/IL-6/PGE2 M-MDSC at the 1:8 cell ratio.

In addition to the phenotypic criteria, M-MDSC should display suppressive properties in one of the suggested assays (9). Except for DC, all cells suppressed significantly the proliferation of PHA-stimulated PBMC at higher cell-to-cell ratios, as compared to PHA/PBMC alone (Figures 2A,B). Thereby, GM-CSF/IL-6/PGE2 M-MDSC displayed the strongest effect, and GM-CSF/IL-4/PGE2-induced M-MDSC displayed the weakest suppression (51.4 ± 8.4% and 24.6 ± 4.6% suppression at 1:8 M-MDSC:PBMC cell ratio, respectively). Therefore, according to the phenotypic criteria and functional assays, we decided to further assess the functions of GM-CSF/IL-6-induced M-MDSC *in vitro*, and in particular, how PGE2 affect their tolerogenic capacity.

### PGE2 Induces a Stabile Immature Phenotype of GM-CSF/IL-6 M-MDSC

**Figure 3:**
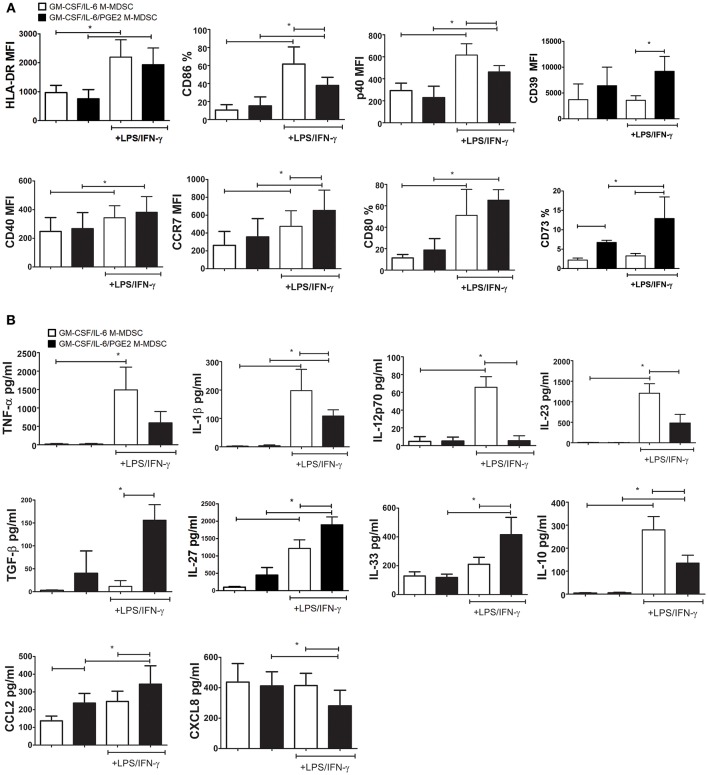
Effects of LPS/IFN-γ on M-MDSC phenotype and cytokines/chemokines production. **(A,B)** The monocytes were cultivated in the presence of GM-CSF/IL-6, or GM-CSF/IL-6/PGE2 for 5 days, and then stimulated with LPS/IFN-γ, or left unstimulated, for the next 16 h. **(A)** The results on surface (HLA-DR, CD86, CD40, CD39, CD73, and CD80) and intracellular (CCR7 and p40) expression obtained by flow cytometry are presented as mean % or mean fluorescence intensity (MFI) ± SD from 4 independent experiments (or 3 experiments in case of CD39 and CD73). **(B)** The levels cytokines/chemokines in cell-free supernatants from those cultures were analyzed by ELISA or beads-based immunoassay and the results are shown as mean pg/ml ± SD (*n* = 4). ^*^p < 0.05 as indicated by the line (RM ANOVA, Tukey post-test).

The stimulation of M-MDSC with IFN-γ and TLR-4 agonists was shown to up-regulate their MHC class II expression and the capacity for Treg inductions (24, 49, 50). Therefore, we tested the phenotype of GM-CSF/IL-6 M-MDSC and GM-CSF/IL-6/PGE2 M-MDSC and cytokines production of M-MDSC stimulated with LPS/IFN-γ or unstimulated. The doses of LPS and IFN-γ applied for the stimulation of M-MDSC did not affect significantly their apoptosis (Supplementary Figure 1). Without the stimulation, both GM-CSF/IL-6 M-MDSC and GM-CSF/IL-6/PGE2 M-MDSC displayed low surface expression of HLA-DR, CD86, CD80, and intracellular expression of CCR7 and p40 subunit. However, GM-CSF/IL-6/PGE2 M-MDSC contained a higher percentage of CD73^+^ cells compared to GM-CSF/IL-6 M-MDSC. Such a difference was found within both HLA-DR^−^/CD14^+^ and HLA-DR^+^/CD14^+^ subsets of non-stimulated M-MDSC (Supplementary Figure 2). After the stimulation with LPS/IFN-γ, both M-MDSC types upregulated significantly HLA-DR, CD80, CD40, and CCR7 expression. Thereby, GM-CSF/IL-6/PGE2 M-MDSC displayed significantly higher CCR7, CD39, and CD73 expression compared to GM-CSF/IL-6 M-MDSC. In contrast, GM-CSF/IL-6 M-MDSC stimulated with LPS/IFN-γ also upregulated significantly CD86 and p40 subunit, unlike LPS/IFN-γ-treated GM-CSF/IL-6/PGE2 M-MDSC (Figure 3A). Therefore, PGE2 reduced the maturation capacity of GM-CSF/IL-6 M-MDSC.

The analysis of cytokines production by GM-CSF/IL-6 M-MDSC and GM-CSF/IL-6/PGE2 M-MDSC suggested significant differences between these cells, but predominantly after the LPS/IFN-γ stimulation. GM-CSF/IL-6/PGE2 M-MDSC displayed a significantly lower capacity to produce TNF-α, IL-1β, IL-12p70, and IL-23 after the stimulation with LPS/IFN-γ, compared to GM-CSF/IL-6 M-MDSC (Figure 3B). These cells also displayed a lower capacity to produce IL-10 compared to GM-CSF/IL-6 M-MDSC. In contrast, GM-CSF/IL-6/PGE2 M-MDSC produced significantly more TGF-β, IL-27, and IL-33. By analyzing chemokines, we found that GM-CSF/IL-6/PGE2 M-MDSC produced significantly more CCL2, both in the presence and absence of stimulation with LPS/IFN-γ, as well as less CXCL8 after the stimulation. Cumulatively, these results suggested that GM-CSF/IL-6 M-MDSC and GM-CSF/IL-6/PGE2 M-MDSC stimulated with LPS/IFN-γ differed significantly in their functional potential and could possibly display different Th polarization capacity in co-culture with T cells.

### PGE2 Potentiates Suppressive Effects of GM-CSF/IL-6 M-MDSC on T Cells and Promote Their Th2 Polarization

**Figure 4:**
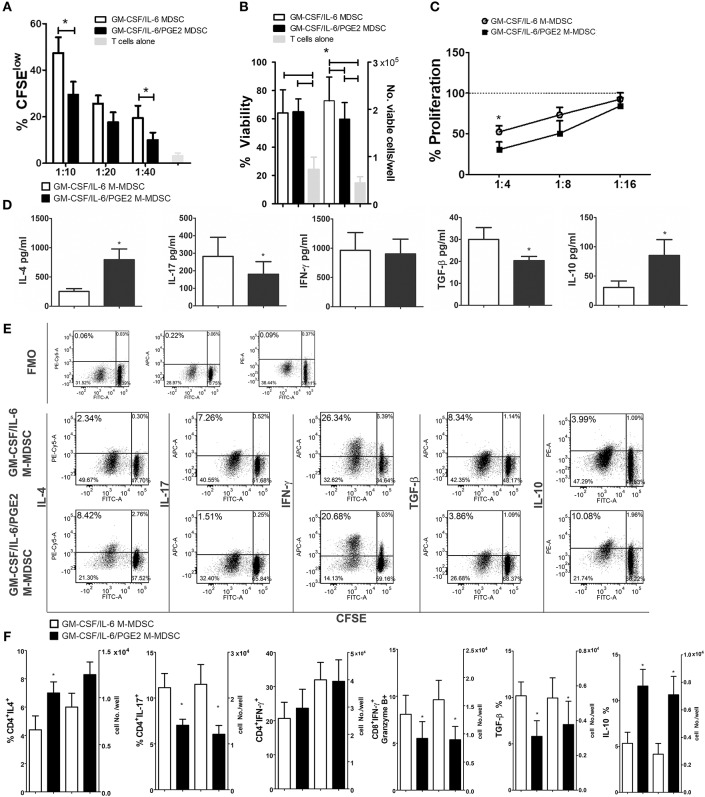
Effects of LPS/IFN-γ-stimulated M-MDSC on proliferation and differentiation of allogeneic T cells. **(A)** The proliferation of MACS-purified allogeneic CFSE-labeled T cells (1 × 10^5^/well) in the presence or absence of different number of LPS/IFN-γ-stimulated M-MDSC (1 × 10^4^-0.25 × 10^4^/well) was determined by flow cytometry after 5 days of co-cultivation, and the results from one representative experiment are shown as mean proliferation % ± SD of triplicates. **(B)** The viability and cell number of the T cells/well was determined on Muse Cell Analyzer, as described, and the data is presented as mean ± SD of 5 independent experiments. **(C)** The proliferation of allogeneic T cells in the presence of CD3/CD28 stimulation and different number of LPS/IFN-γ-stimulated M-MDSC (2.5–0.62 × 10^4^/well) was determined by flow cytometry after 5 days of cultivation, and the results are shown as mean relative proliferation ± SD, i.e., % proliferation of control CD3/CD28-stimulated T cells (100%) from 3 independent experiments. **(A,B)** ^*^p < 0.05 GM-CSF/IL-6 M-MDSC vs. corresponding GM-CSF/IL-6/PGE2 M-MDSC (RM ANOVA, Tukey post-test). **(D)** The levels of indicated cytokines, shown as mean pg/ml ± SD, were determined from the supernatants of 1:10 M-MDSC/T cell co-cultures carried out as in **(A)** and treated for 4 h with PMA/Ca ionophore. The levels of cytokines were standardized to 1 × 10^5^ of viable T cells from the co-cultures. **(E)** Expression of intracellular cytokines was determined within CFSE-labeled T cells co-cultivated at 1:10 cell ratio as in **(A)** and treated for 3 h with PMA/ionomycin/monensin. Data from one representative experiment are shown. **(F)** The percentages and cell number of CD4^+^ IFN-γ^+^, CD4^+^ IL-4, CD4^+^ IL-17, CD8^+^ IFN-γ^+^ Granzyme B^+^, TGF-β^+^, and IL-10^+^cells were determined by flow cytometry from the M-MDSC/T cell co-cultures carried out at 1:10 cell-to-cell ratios as in **(A)** and treated for 3 h with PMA/ionomycin/monensin. The cell number was calculated from the absolute number of viable T cells after the cultures **(B)** and % of positive cells from flow cytometry. ^*^p < 0.05 paired *T*-test.

To investigate how the results on M-MDSC phenotype and cytokines production correlate with the Th polarization capacity of these cells, we used LPS/IFN-γ-stimulated M-MDSC for cultivation with MACS-purified allogeneic T cells. Both GM-CSF/IL-6 M-MDSC and GM-CSF/IL-6/PGE2 M-MDSC were able to induce alloreactive T cell proliferation after the stimulation (Figure 4A), although the response was much lower than the T-cell proliferation induced by LPS/IFN-γ-treated DC (data not shown). However, GM-CSF/IL-6/PGE2 M-MDSC displayed a significantly lower capacity to induce alloreactive T cells proliferation, compared to GM-CSF/IL-6 M-MDSC (Figure 4A). The viability of T cells in co-cultures did not differ significantly, so the number of viable T cells in co-cultures with GM-CSF/IL-6 M-MDSC was higher than in co-cultures with GM-CSF/IL-6/PGE2 M-MDSC (Figure 4B). In the suppressive assay with CD3/CD28-stimulated allogeneic T cells, GM-CSF/IL-6/PGE2 M-MDSC displayed significantly stronger capacity to suppress the proliferation of T cells, compared to GM-CSF/IL-6 M-MDSC, especially when higher number of M-MDSC was present in the co-cultures (1:4, M-MDSC:T cell ratio, respectively) (Figure 4C).

When the cytokines produced in the co-cultures with T cells were analyzed, we found significantly lower levels of IL-17 and TGF-β, and significantly higher levels of IL-4 and IL-10 in the co-cultures with GM-CSF/IL-6/PGE2 M-MDSC, as compared to GM-CSF/IL-6 M-MDSC. In contrast, the levels of IFN-γ were similar between the two M-MDSC type containing co-cultures (Figure 4D). These results were confirmed by analyzing the intracellular levels of cytokines in CFSE-stained T cells from the co-cultures (Figure 4E), in which the most cytokine-producing cells were found within CFSE-low (proliferating) cells. Considering that the T cells cultivated without M-MDSC did not proliferate or display significant levels of intracellular cytokines (Supplementary Figure 3), these results suggested that the allogeneic proliferation was required for cytokine production by T cells. Additionally, we analyzed intracellular cytokines within CD4^+^ and CD8^+^ T subsets and found that a significantly lower percentage of CD4^+^IL-17^+^ T cells, and a higher percentage of CD4^+^IL-4^+^ T cells was induced by GM-CSF/IL-6/PGE2 M-MDSC, as compared to GM-CSF/IL-6 M-MDSC. These results were also confirmed by analyzing the expression of GATA-3 and ROR-γ*t* within CD4^+^ T cells (data not shown). The percentages of IFN-γ^+^CD4^+^ T cells were similar between the tested groups. On the other side, the percentage of IFNγ^+^ Granzyme B^+^ CD8^+^ cytotoxic T cells (CTL) was lower in the co-cultures with GM-CSF/IL-6/PGE2 M-MDSC, as compared to GM-CSF/IL-6 M-MDSC. The differences in the percentages of cytokine-producing cells correlated with the absolute number of cells in these co-cultures (Figure 4F). Therefore, GM-CSF/IL-6/PGE2-induced M-MDSC displayed an increased ratio of Th2/Th17 cells, and a reduced capacity to induce CTL compared to GM-CSF/IL-6 M-MDSC, pointing to their anti-inflammatory polarization potential.

### PGE2 Reduces the Capacity of GM-CSF/IL-6 M-MDSC to Induce Conventional Treg

**Figure 5:**
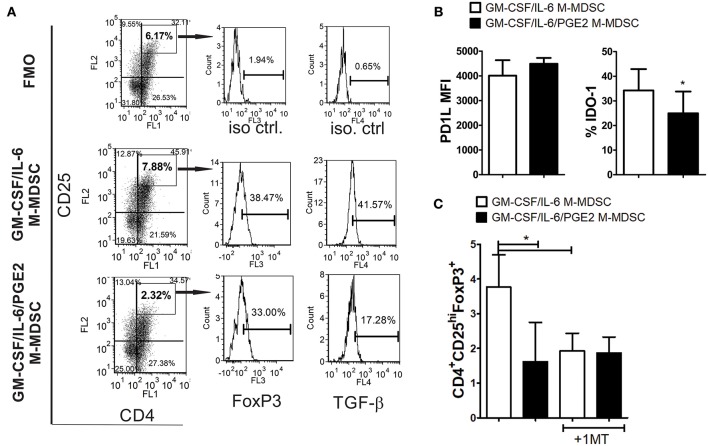
The capacity of LPS-IFN-γ stimulated M-MDSC to induce FoxP3^+^ Treg. **(A)** A representative analysis of FoxP3^+^ Treg is shown from the experiments in which M-MDSC (2 × 10^3^/well) were co-cultivated with allogeneic T cells for 3 days, followed by IL-2 treatment for the next 3 days. The presented histograms of FoxP3 and TGF-β are shown from CD4^+^CD25^hi^ gates, and the markers were set according to FMO control. **(B)** The surface expression of PD-L1 and intracellular expression of IDO-1 were determined by flow cytometry after the staining of LPS/IFN-γ stimulated M-MDSC, and the results are shown as mean MFI or % ± SD of 3 independent experiments. ^*^p < 0.05 paired *T*-test. **(C)** The summarized data are shown on the % of CD4^+^CD25^hi^FoxP3^+^ cells ± SD (*n* = 3) induced in the co-cultures with M-MDSC that were carried out as in **(A)**, either in the presence or absence of 1-MT. ^*^p < 0.05 as indicated by line (RM ANOVA, Tukey post-test).

To study the capacity of GM-CSF/IL-6 M-MDSC and GM-CSF/IL-6/PGE2 M-MDSC to induce conventional Treg, allogeneic T cells were co-cultivated with LPS/IFN-γ-stimulated M-MDSC at 1:50 (MDSC: T cell ratio) for 3 days and then expanded with IL-2, before the analysis of CD4^+^CD25^hi^FoxP3^+^ T cells. These culture conditions enabled a higher viability of T cells and a lower difference in the total cell number between the co-cultures with GM-CSF/IL-6 M-MDSC and GM-CSF/IL-6/PGE2 M-MDSC (Supplementary Figure 4). Interestingly, it was found that GM-CSF/IL-6 M-MDSC induced a significantly higher percentage of CD4^+^CD25^hi^FoxP3^+^ Treg compared to GM-CSF/IL-6/PGE2 M-MDSC (Figure 5A). Similar results were observed when Treg were analyzed as CD4^+^CD25^+^CD127^−^FoxP3^+^ T cells (data not shown). Although the expression of FoxP3 within CD4^+^CD25^hi^ (or CD4^+^CD25^+^CD127^−^) Treg cells was similar between the two groups, TGF-β expression was reduced within Foxp3^+^ Treg co-cultivated with GM-CSF/IL-6/PGE2 M-MDSC. T cells cultivated without M-MDSC contained low percentage of total CD25^+^ cells (2.4 ± 1.3%), and no cells were found within CD4^+^CD25^hi^ region as set for the T cells co-cultivated with M-MDSC (data not shown).

To assess the mechanism by which GM-CSF/IL-6 M-MDSC induce a higher percentage of FoxP3^+^ Treg, the surface expression of PD-L1 and IDO-1 were analyzed, as these are critical molecules for the induction of FoxP3^+^ Treg (31, 51). It was found that PD-L1 was expressed similarly between the two M-MDSC types, whereas the expression of IDO-1 was higher on GM-CSF/IL-6 M-MDSC, as compared to GM-CSF/IL-6/PGE2 M-MDSC (Figure 5B). When IDO-1 inhibitor (1-MT) was used in the co-cultures with T cells, the percentage of FoxP3^+^ Treg induced by GM-CSF/IL-6 M-MDSC and GM-CSF/IL-6/PGE2 MDSC was similarly lower (Figure 5C). These results suggested that increased IDO-1 expression by GM-CSF/IL-6 M-MDSC was responsible for a higher capacity of these cells to induce FoxP3^+^ Treg.

### PGE2 Increases the Capacity of GM-CSF/IL-6 M-MDSC to Induce IL-10 Producing T Cells

**Figure 6:**
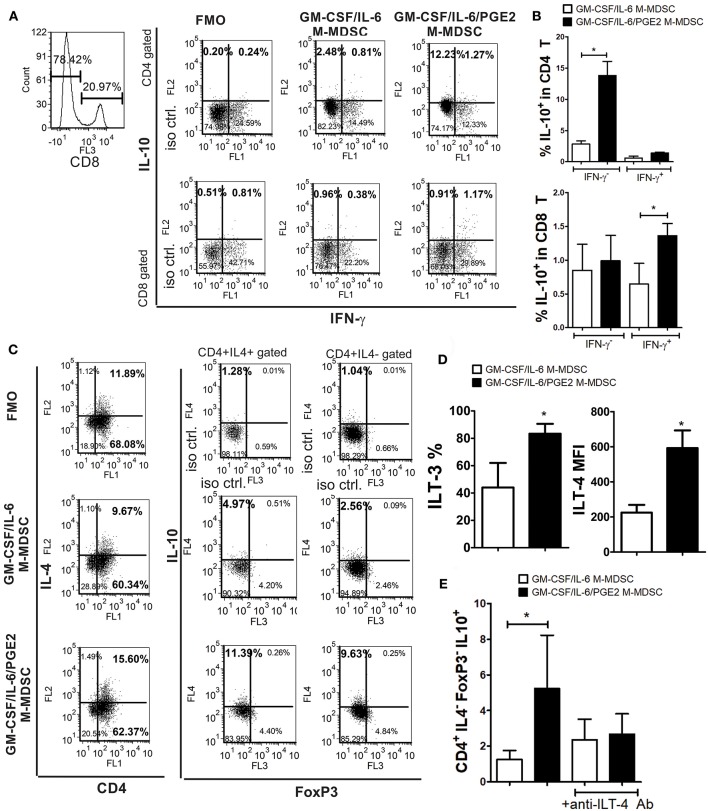
The capacity of LPS/IFN-γ-stimulated M-MDSC to induce IL-10-producing T cells. **(A)** A representative analysis is shown of IL-10 and IFN-γ expression within gated CD8^+^ and CD8^−^(CD4^+^) T cell populations after the co-culture with LPS/IFN-γ-stimulated M-MDSC (2 × 10^3^/well) for 3 days, followed by the IL-2 treatment for additional 3 days. **(B)** The summarized results from 3 independent experiments are shown as % of IL-10^+^ in CD4^+^ or CD8^+^ T cells co-expressing IFN-γ or not. **(C)** A representative analysis of IL-10 and FoxP3 expression within CD4^+^IL-4^+^ and CD4^+^IL4^−^ T cells is shown from the experiments performed as in **(A)**. **(D)** The surface expression of ILT3 and ILT4 were determined by flow cytometry after the staining of LPS/IFN-γ-stimulated M-MDSC and the results are shown as mean MFI or % ± SD of 3 independent experiments. ^*^p < 0.05 paired *T*-test. **(E)** The summarized data are shown on the % of CD4^+^IL-4^−^FoxP3^−^IL10^+^ (Tr-1) cells ± SD (*n* = 3) induced in the co-cultures with M-MDSC that were carried out as in **(A)**, either in the presence of anti-ILT-4 Ab or isotype control Ab. ^*^p < 0.05 as indicated by the line (RM ANOVA, Tukey post-test).

The lower capacity of GM-CSF/IL-6/PGE2 M-MDSC to induce conventional Treg could explain the lower levels of TGF-β found in the co-cultures with GM-CSF/IL-6/PGE2 M-MDSC, but not the increased levels of IL-10 in the same co-cultures. To assess the source of increased IL-10 production by T cells we co-cultivated T cells with MDSC at 1:50 (M-MDSC:T) cell ratio and analyzed the expression of this cytokine within CD4^+^ and CD8^+^ T cell subsets by flow cytometry. Compared to GM-CSF/IL-6 M-MDSC, GM-CSF/IL-6/PGE2 M-MDSC induced significantly higher expression of IL-10 within CD4^+^IFN-γ^−^ T cells, but not within CD4^+^IFN-γ^+^ (Th1) cells (Figures 6A,B). In contrast, GM-CSF/IL-6/PGE2 M-MDSC increased the expression of IL-10 within CD8^+^IFN-γ^+^ T cells, and not within CD8^+^IFN-γ^−^, as compared to GM-CSF/IL-6 M-MDSC. However, the presence of IL-10 in CD8^+^ T cells was much lower than in CD4^+^IFN-γ^−^ T cells.

Therefore, we then analyzed the expression of IL-10 in CD4^+^IL-4^+^ (Th2) and CD4^+^IL-4^−^FoxP3^−^ T cells, also identified as Tr-1 cells (29, 40). We found that GM-CSF/IL-6/PGE2 M-MDSC induced significantly higher expression of IL-10 within both CD4^+^IL-4^+^ and CD4^+^IL-4^−^FoxP3^−^ T cells, as compared to GM-CSF/IL-6 M-MDSC (Figure 6C). In co-cultures with GM-CSF/IL-6/PGE2 M-MDSC, the percentage of IL-10^+^ Th2 cells was 2.1 ± 0.4 (*n* = 3) times higher than in co-cultures with GM-CSF/IL-6 M-MDSC, whereas the percentage of Tr-1 cells increased even higher [3.2 ± 0.6 (*n* = 3)]. Moreover, the total number of CD4^+^IL4^−^FoxP3^−^ cells was about 4 times higher than the number of Th2 cells, suggesting that Tr-1 cells contributed more to the total number of IL-10^+^ T cells.

To analyze the mechanisms responsible for the Tr-1 induction by GM-CSF/IL-6/PGE2 M-MDSC, we focused on ILT-3 and ILT-4 expression, since these molecules were found critical for the induction of these cells (29, 30). Flow cytometry analysis showed that GM-CSF/IL-6/PGE2 M-MDSC expressed significantly more ILT-3 and ILT-4, as compared to GM-CSF/IL-6 M-MDSC (Figure 6D). Additionally, when M-MDSC/T cell co-cultures were carried out in the presence of blocking anti-ILT-4 Ab (Figure 6E) or anti-ILT-3 Ab (data not shown), the percentage of Tr-1 cells in the co-cultures with GM-CSF/IL-6/PGE2 M-MDSC was similar to the percentage Tr-1 cells induced in the co-cultures with GM-CSF/IL-6 M-MDSC. These results suggested that GM-CSF/IL-6/PGE2 M-MDSC utilize ILT-3- and ILT-4-dependent mechanisms to induce the development of Tr-1 cells.

## Discussion

MDSC, particularly M-MDSC, have been recognized as a major limiting factor for the efficacy of checkpoint inhibition therapy (2, 4, 5). A probable cause includes many mechanisms by which they suppress the activation of T cells (3, 10, 11) and induce FoxP3^+^ Treg (23). The relative contribution of these mechanisms, especially in the induction of different Treg subsets, remained poorly investigated. To facilitate such an inquiry, we have developed an original model for the generation of M-MDSC and demonstrated that the monocytes differentiated in the presence of GM-CSF and IL-6 acquire M-MDSC phenotype, produce IL-10, exhibit suppressive properties, and induce a higher percentage of FoxP3^+^ Treg via IDO-1-dependent mechanisms. PGE2, a key factor produced in chronic inflammation and tumor (44), potentiate GM-CSF/IL-6-dependent induction of M-MDSC, their suppressive potential and their capacity to induce the Th2 response *in vitro*. Moreover, we showed for the first time that PGE2 does not increase the capacity of GM-CSF/IL-6 M-MDSC to induce CD4^+^CD25^hi^FoxP3^+^Treg, but rather augment their capacity to induce IL-10 production by CD8^+^ IFN-γ^+^ T cells, Th2 cells, and particularly, by CD4^+^ IL-4^−^ FoxP3^−^ Tr-1 cells via ILT-4 and ILT-3 dependent mechanisms.

Previous reports suggested that M-MDSC could be differentiated *in vitro* from monocytes by using GM-CSF, IL-4, and PGE2 (36, 38). Our data confirmed that this protocol induces suppressive cells (especially compared to non-suppressive DC) with low CD1a expression and high CD14 expression. The described mechanisms behind the suppressive activity of GM-CSF/IL-4/PGE2 M-MDSC include EP2/EP4-dependent positive feedback loop between PGE2 and COX2, which drives an increased expression of suppressive markers on these M-MDSC, such as PD-L1 (36). However, unlike Obermajer et al. (36), the GM-CSF/IL-4/PGE2 M-MDSC obtained in our experiments expressed CD209. This could be due to different basal media used and lower doses of GM-CSF used in their experiments. DC-SIGN (CD209) is a hallmark of IL-4-induced effects on DC, and its expression is down-regulated in the presence of TGF-β (52). Unlike the other authors which used TGF-β-containing fetal calf serum (36, 37, 53), we used serum-free medium, which could be a probable reason for detecting a higher level of CD209 on GM-CSF/IL-4/PGE2 induced M-MDSC. Moreover, the percentage of HLA-DR^−^CD14^+^CD33^+^CD11b^+^ cells induced by GM-CSF/IL-4/PGE2, and the suppressive capacity of these cells, was lower than those induced by using GM-CSF/IL-6 or GM-CSF/IL-6/PGE2 cocktail.

GM-CSF/IL-6-based protocols induced much lower percentage CD209^+^ cells, and in the absence of PGE2, GM-CSF, and IL-6 induced a higher expression of CD206. Although more specific in mice, some studies suggested that CD206 is a marker of human M2 macrophages generated from monocytes in the presence of M-CSF, but not in the presence of GM-CSF knowing to induce M1 type macrophages (54, 55). In contrast, other reports suggested that high expression of CD209 better correlates with the M2 phenotype of human macrophages (56). M-MDSC in our experiments were heterogeneous, and according to their phenotype and cytokines production, they hardly fit into the standard M1/M2 macrophage model. Namely, GM-CSF/IL-6-induced M-MDSC displayed a higher capacity to up-regulate CD86, p40, a subunit of IL-12/IL-23 (57), and proinflammatory cytokines TNF-α, IL-1β, IL-12, and IL-23, as well as a lower capacity to produce TGF-β in comparison to GM-CSF/IL-6/PGE2 M-MDSC. These properties resemble more to M1-like properties of GM-CSF/IL-6-induced M-MDSC. However, GM-CSF/IL-6 M-MDSC also produced increased levels of IL-10 and displayed suppressive capacity in the co-cultures with allogeneic PBMC and T cells, all of which mark M2 type macrophages (54, 55, 58). Unlike the standard protocols for macrophages differentiation (54–56), we used IL-6 and IL-6/PGE2 from the beginning of differentiation, and about 23% and 43% of these cells, respectively, lacked the expression of HLA-DR, which is the main reason for designating them as M-MDSC, rather than macrophages. It is not clear at the moment whether additional stages of myeloid cell differentiation are present in our M-MDSC population and to which extent. Therefore, additional phenotypic and functional characterization of *in vitro* induced M-MDSC are necessary to assess the heterogenicity of these cells and relate them to their *in vivo* counterparts.

The combination of GM-CSF and IL-6 was shown to potentiate the generation of IL-4Rα^+^ MDSC from bone marrow in mice and humans (59). Additionally, by analyzing the tumor-produced factors which induce suppressive CD33^+^ cells from PBMC, Lechner et al. (35) demonstrated that the combination of GM-CSF and IL-6 is more potent than the combination of GM-CSF and other mediators, such as PGE2. Although these authors suggested that GM-CSF/IL-6 induced CD33^+^ cells were mononuclear, the described phenotype was CD11b^+^CD66b^+^HLA-DR^low^IL-13Ra2^int^, which along with their increased NADPH oxidase activity (35), corresponds to PMN-MDSC (9, 60). It was left unclear, whether M-MDSC also contribute to the suppressive effects of CD33^+^ cells induced by GM-CSF and IL-6. To our knowledge, this is the first report showing the ability of GM-CSF/IL-6 combination to generate suppressive HLA-DR^−/low^CD14^+^CD33^+^CD11b^+^ M-MDSC as well. Moreover, PGE2 potentiated significantly this induction. We found significantly higher percentage of CD39^+^CD73^+^ M-MDSC in cultures with GM-CSF/IL-6/PGE2 compared to GM-CSF/IL-6 alone. These molecules are involved in the adenosine-generating pathway, which was found critical for an increased infiltration and suppressive functions MDSC and tumor associated macrophages in cancer patients (61, 62). The fact that M-MDSC induced in presence of PGE2 contained a higher percentage of CD39^+^CD73^+^ cells within both HLA-DR^−/low^ and HLA-DR^+^ subsets, suggest that both populations contribute to the suppressive activity these cells. M-MDSC were shown to accumulate in tumor via CCL2-dependent mechanisms (63), whereas PMN-MDSC accumulate in a CXCL8-depenent manner (64). The phenomenon that PGE2 potentiates the production of CCL2 and simultaneously reduces CXCL8 production by GM-CSF/IL-6 M-MDSC, could explain the observation that M-MDSC accumulate preferentially in PGE2-rich tumor site, rather than PMN-MDSC (3, 11), although this hypothesis needs to be tested independently.

Significant functional differences were found between GM-CSF/IL-6 M-MDSC and GM-CSF/IL-6/PGE2 M-MDSC after their stimulation with LPS/IFN-γ, which could explain differences in their allostimulatory capacity, Th polarization, and Treg induction. IFN-γ and TLR4 agonists were shown to activate NF-kB signaling in MDSC, up-regulate their MHC class II expression, and significantly contribute to the suppressive functions of MDSC in contact with T cells (24, 49, 50, 65). In respect to this, a lower allostimulatory capacity of LPS/IFN-γ-stimulated GM-CSF/IL-6/PGE2 M-MDSC could be explained by their lower capacity to up-regulate CD86 expression and pro-inflammatory cytokines, as well as a higher capacity to produce TGF-β. TGF-β was shown to exhibit direct anti-proliferative effects on T cells (66), and has a critical role in cancer driven immunosuppression (67). Moreover, an increased TGF-β production by CD14^+^HLA-DR^low/−^ M-MDSCs from patients with melanoma was shown to be PGE2 and COX2 dependent (18).

In contrast, GM-CSF/IL-6 M-MDSC produced higher levels of IL-10, which together with the up-regulated CD80 expression could have contributed to their increased capacity for inducing CD4^+^CD25^hi^FoxP3^+^ Treg. Although CD80 can act as a co-stimulatory molecule, it ligates CTLA-4 with a higher affinity than CD28 (68). Accordingly, in an ovarian carcinoma-bearing mice model, IFN-γ-stimulated M-MDSC increased MHC class II, CD80, and IL-10 expression, and induced CD4^+^CD25^+^ Treg in a CTLA-4/CD80-dependent manner (65), which is in line with our results on IL-10-producing GM-CSF/IL-6 M-MDSC. Additionally, we found that GM-CSF/IL-6 M-MDSC display a higher IDO-1 expression, which could be involved directly in the induction of a higher percentage of CD4^+^CD25^hi^FoxP3^+^ Treg by these cells, compared to GM-CSF/IL-6/PGE2. LPS and IFN-γ are strong inducers of IDO-1 (69) and IL-10 was shown to significantly potentiate IFN-γ-mediated IDO-1 expression (70). Therefore, a higher capacity of GM-CSF/IL-6 M-MDSC to produce IL-10, compared to GM-CSF/IL-6/PGE2, could be a reason for their higher expression of IDO-1. Other findings (69), including our own with nanomaterials or parasite products induced tolerogenic DC (31, 71–73), showed that FoxP3^+^ cells induced via IDO-1-dependent mechanisms express CD39, CD73, and TGF-β, which contribute to their suppressive functions in contact with allogeneic T cells. However, it should be noted that in the presence of IDO-1 inhibitor (1-MT), both M-MDSC types induced about 2% of FoxP3^+^ Treg. These results suggest that both M-MDSC types possess additional mechanisms by which they induce CD4^+^FoxP3^+^ Treg, independent of IDO-1.

Interestingly, GM-CSF/IL-6/PGE2 M-MDSC displayed a higher suppressive capacity in the co-culture with CD3/CD28-stimulated T cells, without inducing an increased percentage of FoxP3^+^ Treg. A probable reason for this finding is a higher capacity of these cells to induce IL-10-producing Th2 and Tr-1 cells. PGE2 potentiated IL-33 production by GM-CSF/IL-6 induced M-MDSC. This cytokine is highly produced by necrotic tumor cells as an alarmin, and its role in driving the recruitment and activation of MDSC was shown previously (74). Although IL-33 production by M-MDSC was not shown before, endogenous PGE2 was reported to amplify IL-33 production by macrophages via EP2/EP4 cAMP-dependent pathway (75). Therefore, it is possible that similar mechanisms were involved in a higher capacity of GM-CSF/IL-6/PGE2 M-MDSC to produce IL-33. IL-33 is a potent inducer of Th2 cell differentiation and their maintenance, acting via ST-2 receptor (76). In line with this, we showed an increased capacity of IL-33-producing GM-CSF/IL-6/PGE2 M-MDSC to induce Th2 cells. Th2 cells were shown to promote tumor development, unlike Th1 cells specific for the same antigens (77), suggesting that GM-CSF/IL-6/PGE2 M-MDSC cells induced in our model display the functional resemblance to M-MDSC *in vivo* (7).

A lower percentage of IFN-γ^+^ Granzyme B^+^ CTL in co-culture with GM-CSF/IL-6/PGE2 M-MDSC could be explained by a lower capacity of these cells to produce IL-12, as this cytokine is critical for CLT induction (78). In spite of this, we did not observe down-regulation of IFN-γ production by Th1 cells, and these cells did not produce a significant amount of IL-10. A possible explanation of this phenomenon could be a higher capacity of GM-CSF/IL-6/PGE2 M-MDSC to produce IL-27. Namely, this cytokine was shown to promote the differentiation of Th1 cells, but also to inhibit directly the development of Th17 cells (79). Therefore, both lower IL-23 production and increased IL-27 could explain the down-regulated capacity of GM-CSF/IL-6/PGE2 MDSC to induce Th17 cells, compared to GM-CSF/IL-6 M-MDSC. To our knowledge, this is the first report showing the production of IL-27 by M-MDSC, but the significance of this finding is still not clear. The role of IL-27 in cancer is still a matter of debate, considering that both pro-inflammatory and anti-inflammatory actions of IL-27 were demonstrated (80). In relation to our results, IL-27 was shown as an important inducer of Tr-1 cells, particularly in the presence of TGF-β (81). Moreover, it was reported that Tr1 cells can make up to 30% of all tumor-infiltrating lymphocytes in some tumors (33). Therefore, the roles of IL-27 and TGF-β produced by M-MDSC in the tumor microenvironment deserves further investigations.

In our study, Tr-1 cells were phenotypically identified as CD4^+^FoxP3^−^IL-4^−^IL-10^+^ according to other reports (29, 40). Moreover, in line with other findings on tolerogenic DC (40, 82), we showed that the induction of these cells is ILT3- and ILT-4-dependent. PGE2 was shown previously to increase the expression of ILT4 and ILT3 by M-MDSC (36), but this is the first report to explain the role of these molecules in the induction of Tr-1 cells. Moreover, we showed previously that blockage of ILT-3 and ILT-4 on tolerogenic DC induced by cellulose nanomaterials (31) or mesenchymal stem cells (83) reduced both the percentage of induced Tr-1 cells, as well as the suppressive capacity of total T cell population containing Tr-1 cells. As before, it should be noted that even after blocking of ILT3 and ILT4, both M-MDSC types induced a low percentage of Tr-1 cells, suggesting that additional mechanisms could be involved in the induction of Tr1 cells by M-MDSC. Several studies suggested that Tr1 cells could induce stronger suppressive effects *in vivo*, compared to FoxP3^+^ Treg, due to their actions in both antigen-specific and antigen non-specific manner (33, 34). These results could also explain a higher *in vitro* suppressive capacity of GM-CSF/IL-6/PGE2 M-MDSC, as compared to GM-CSF/IL-6 M-MDSC. In addition, our preliminary experiments in a rat model of experimental autoimmune encephalomyelitis (EAE) suggested that the application of both GM-CSF/IL-6- and GM-CSF/IL-6/PGE2-induced bone-marrow cells can suppress the development of EAE symptoms, and the duration of the disease. However, it remained to be investigated whether similar mechanisms of FoxP3^+^ and Tr-1 induction are involved in the observed *in vivo* effects. Besides, it should be investigated what is the potential of these M-MDSC in allogeneic transplantation models and whether similar mechanisms of immune suppression could be induced. These studies could provide important clues to which tolerogenic mechanisms should be targeted by *in vitro* generated M-MDSC in the development of a specific immunotherapy for autoimmunity and transplantation therapy.

In conclusion, we found that the combination of GM-CSF and IL-6 induce differentiation of monocytes into a heterogeneous population of M-MDSC which induce TGF-β-producing FoxP3^+^ Treg. PGE2 potentiated the suppressive phenotype and functions of GM-CSF/IL-6 induced M-MDSC and augmented their potential to induce IL-10-producing T cells, including Tr-1 cells. The mechanisms involved in these processes include, but are not limited to, IDO-1, ILT3, and ILT4, which represent potentially promising checkpoint inhibitors in cancer immunotherapy. The described model of human M-MDSC seems a good platform to study novel therapeutic strategies focused on M-MDSC-related effects in tumors and autoimmune diseases.

## Data Availability

All datasets generated for this study are included in the manuscript and/or the supplementary files.

## Author Contributions

MČ, DV, and ST designed the study. ST, BJ, MV, MB, MM, and DV performed the experiments. ST and DV analyzed and interpreted the data. ST wrote the manuscript. MČ and DV supplied the materials and infrastructure. All authors revised critically and approved the manuscript, and agreed to be accountable for all aspects of the work in ensuring that questions related to the accuracy or integrity of any part of the work are appropriately investigated and resolved.

### Conflict of Interest Statement

The authors declare that the research was conducted in the absence of any commercial or financial relationships that could be construed as a potential conflict of interest.
