# Supplementary material for: Prostaglanin-E2 Potentiates the Suppressive Functions of Human Mononuclear Myeloid-Derived Suppressor Cells and Increases Their Capacity to Expand IL-10-Producing Regulatory T Cell Subsets
Source: Front Immunol. 2019 Mar 18;10:475. doi: 10.3389/fimmu.2019.00475 (PMC6431635; doi:10.3389/fimmu.2019.00475)
Supplement: Supplementary file 1 [file Data_Sheet_1.PDF]

Supplementary Material

Supplementary Figures

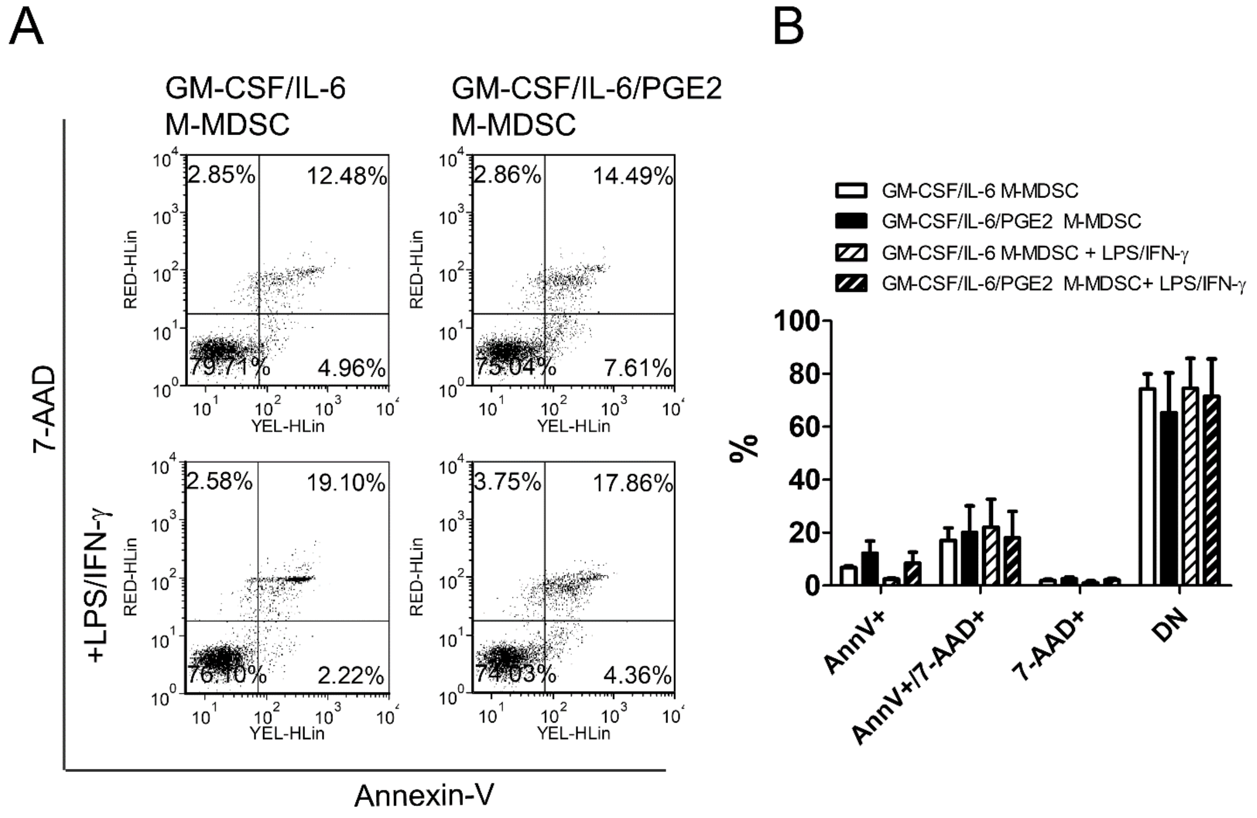

Supplementary Figure 1. Apoptosis of M-MDSC after the cultures and stimulation with LPS/IFN-γ. MACS purified monocytes were cultivated in the presence of GM-CSF/IL-6 or GM-CSF/IL-6/PGE2 for 5 days, followed stimulation with LPS/IFN-γ for 16h. Apoptosis of the harvested cells were analyzed after staining the cells with Annexin-V and 7-AAD, as described in Materials and Methods. **A)** The analysis from a representative experiment is shown, and **B)** the summarized data for early

apoptotic (AnnV+), late apoptotic (AnnV+/7AAD+), necrotic (7-AAD+) and live (DN-double negative) cells from 3 independent experiments is shown as mean ± SD.

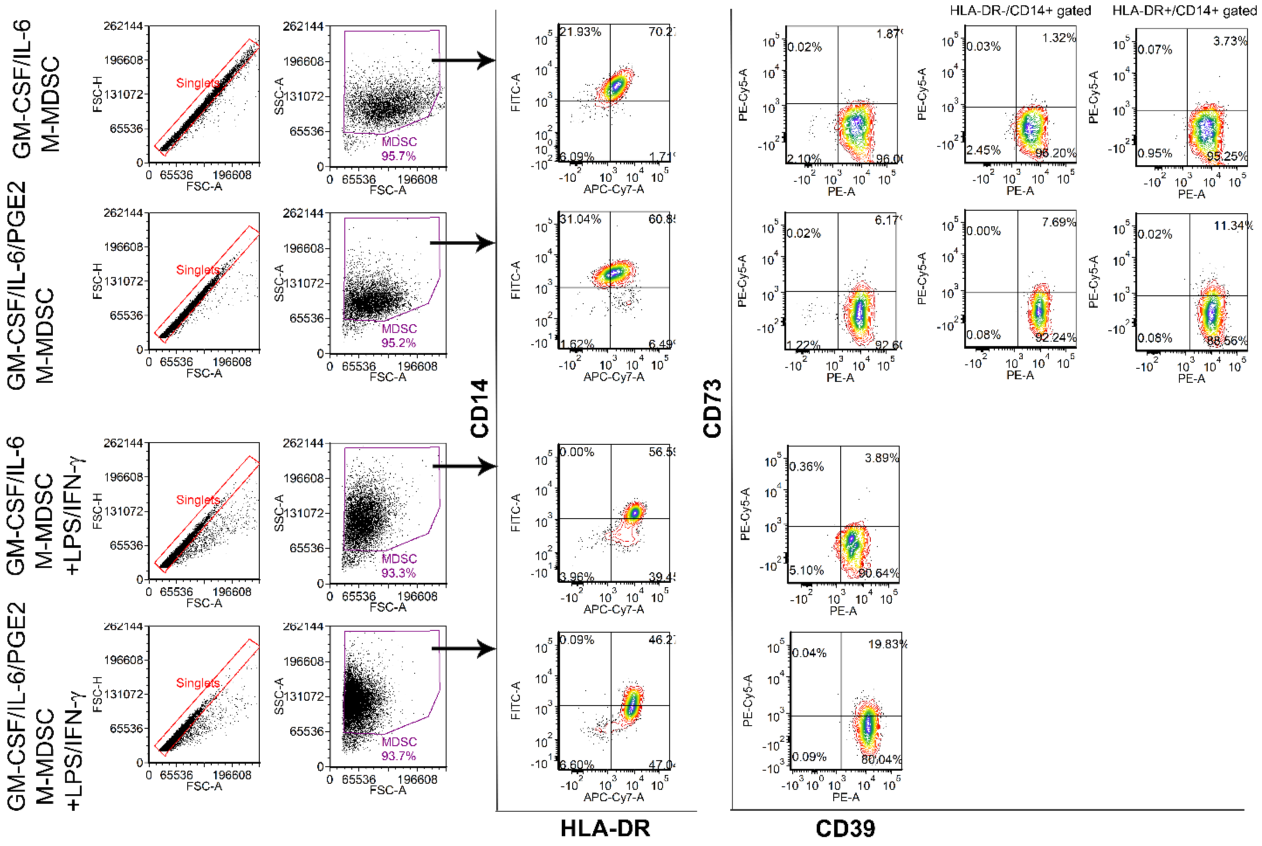

Supplementary Figure 2. Phenotypic analysis of CD73 and CD39 expression of M-MDSC. MACS purified monocytes were cultivated in the presence of GM-CSF/IL-6 or GM-CSF/IL-6/PGE2 for 5 days, followed stimulation with LPS/IFN-γ for 16h. A representative gating strategy is shown and the analysis of CD73/CD39 expression within the total population of M-MDSC obtained, or within the HLA-DR-/CD14+ and HLA-DR+/CD14+ subsets of non-stimulated M-MDSC. The summarized data is shown in Figure 3 of the paper.

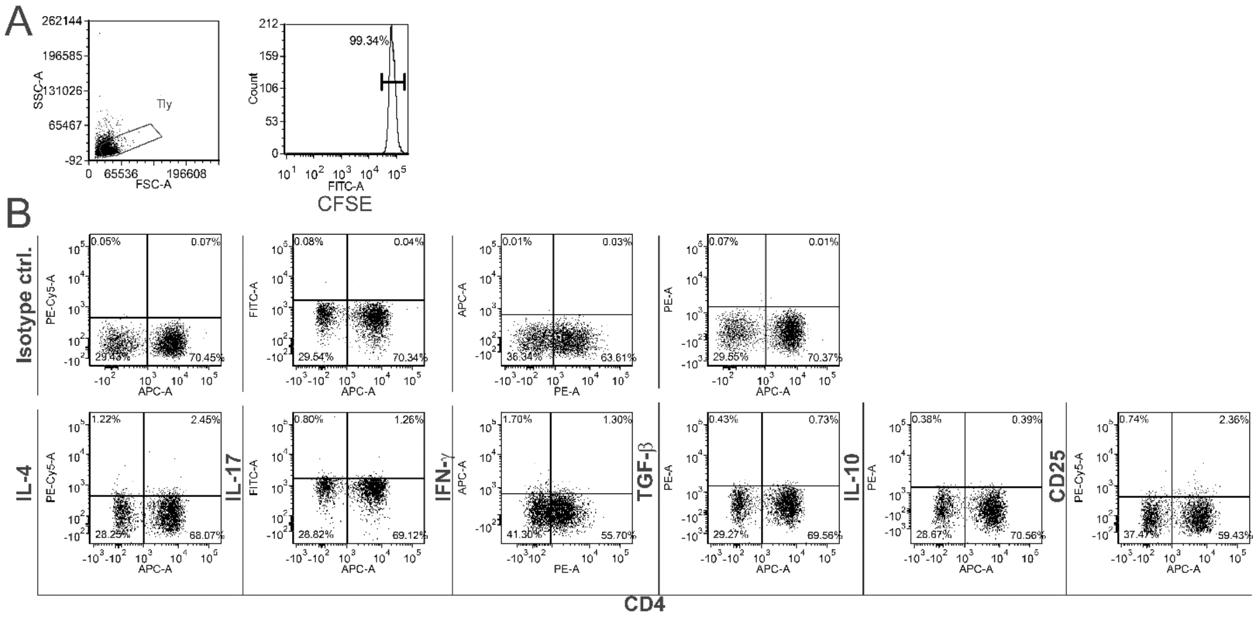

Supplementary Figure 3. A representative analysis of the proliferation and cytokines expression by T cells that were cultivated in the absence of M-MDSC for 3 days, and additional 3 days in the presence of IL-2 (3ng/ml). **A)** The proliferation according to CFSE dilution is shown. **B)** Intracellular cytokines expression of IL-4, IL-17, IFN-γ, TGF-β and IL-10 within T cells, and the surface expression of CD4 and CD25 was analysed after the 6-days cultures and the stimulation of T cells with PMA/Ca ionophore/monensin, as described in Materials and Methods. The % of cytokine-positive cells was determined according to non-specific binding of isotype control Ab, as indicated. The expression of FoxP3 was analyzed within CD25^{hi} CD4+ gate (see Figure 5), but T cells cultivated without MDSC lacked comparable cell population.

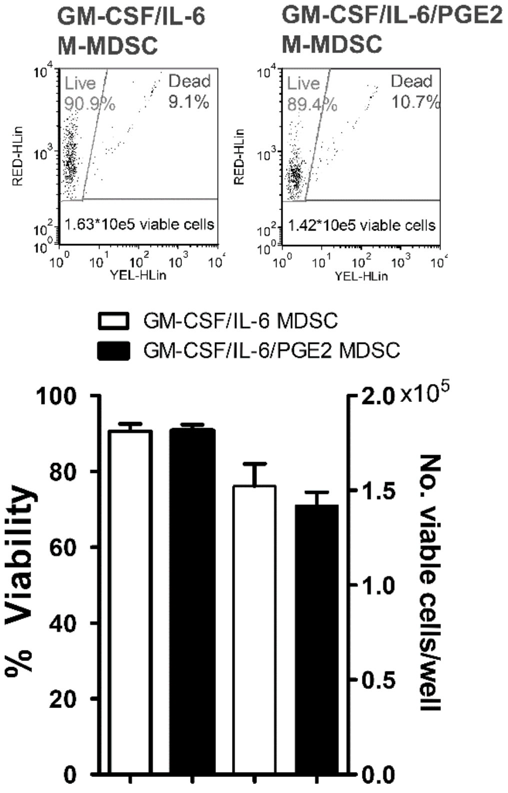

Supplementary Figure 4. The analysis of T cells viability and cell number after the co-culture with M-MDSC. T cells were co-cultivated with M-MDSC at 1:50 (M-MDSC: T cell) ratio for 3 days, followed by treatment with IL-2 for additional 3 days. The viability and cell count were determined on Muse Cell Analyzer, as described in Materials and Methods. A representative data is shown with % of live and dead cells, as well as the total number of viable cells detected per well, and the summarized data collected from 3 independent experiments is shown below as mean ± SD.
